# Discrimination between the effects of pulsed electrical stimulation and electrochemically conditioned medium on human osteoblasts

**DOI:** 10.1186/s13036-023-00393-1

**Published:** 2023-11-23

**Authors:** Meike Bielfeldt, Kai Budde-Sagert, Nikolai Weis, Maren Buenning, Susanne Staehlke, Julius Zimmermann, Nils Arbeiter, Sahba Mobini, María Ujué González, Henrike Rebl, Adelinde Uhrmacher, Ursula van Rienen, Barbara Nebe

**Affiliations:** 1 Institute for Cell Biology, Rostock University Medical Center, 18057 Rostock, Germany; 2 Institute of Communications Engineering, University of Rostock, 18051 Rostock, Germany; 3 Institute for Visual and Analytic Computing, University of Rostock, 18051 Rostock, Germany; 4 Institute of General Electrical Engineering, University of Rostock, 18051 Rostock, Germany; 5 Instituto de Micro y Nanotecnología, IMN-CNM, CSIC (CEI UAM+CSIC), Isaac Newton 8, E-28760 Tres Cantos, Madrid, Spain; 6 Interdisciplinary Faculty, University of Rostock, 18051 Rostock, Germany

**Keywords:** Electrical stimulation, Alternating current, Osteoblasts, ROS, Calcium ions

## Abstract

**Background:**

Electrical stimulation is used for enhanced bone fracture healing. Electrochemical processes occur during the electrical stimulation at the electrodes and influence cellular reactions. Our approach aimed to distinguish between electrochemical and electric field effects on osteoblast-like MG-63 cells. We applied 20 Hz biphasic pulses via platinum electrodes for 2 h. The electrical stimulation of the cell culture medium and subsequent application to cells was compared to directly stimulated cells. The electric field distribution was predicted using a digital twin.

**Results:**

Cyclic voltammetry and electrochemical impedance spectroscopy revealed partial electrolysis at the electrodes, which was confirmed by increased concentrations of hydrogen peroxide in the medium. While both direct stimulation and AC-conditioned medium decreased cell adhesion and spreading, only the direct stimulation enhanced the intracellular calcium ions and reactive oxygen species.

**Conclusion:**

The electrochemical by-product hydrogen peroxide is not the main contributor to the cellular effects of electrical stimulation. However, undesired effects like decreased adhesion are mediated through electrochemical products in stimulated medium. Detailed characterisation and monitoring of the stimulation set up and electrochemical reactions are necessary to find safe electrical stimulation protocols.

**Supplementary Information:**

The online version contains supplementary material available at 10.1186/s13036-023-00393-1.

## Background

The need for long-lasting orthopaedic implants is increasing with the ageing world population. New implant designs strive to increase osseointegration for longer durability. Conductive or piezoelectric biomaterials are under development to enhance bone healing and tissue regeneration [1]. The usage of electrical stimulation (ES) for bone tissue engineering started with the discovery that bone itself has piezoelectric properties [2] and bone formation is seen in areas of negative potentials [3].

Electrical stimulation for bone regeneration can be divided into three modalities. With direct ES, electrodes are implanted directly at the site of damage and ES is delivered via a subcutaneous or external power source. The second method is capacitive coupling, which uses electrodes on the skin on both sides of the fracture with the advantages of not requiring surgical intervention nor charge transfer. The third modality is inductive coupling, where pulsed electromagnetic fields are applied externally [4, 5].

Pre-clinical trials have revealed that the three approaches contributed to enhanced osteogenesis in small animals. However, in large animal models, only direct ES was described to be successful [6]. Clinical studies have observed varying outcomes of enhanced fracture healing but randomised controlled trials are needed to evaluate possible benefits [5, 7]. The discrepancy between these studies comes from different ES modalities as well as the lack of standardised parameters, evaluation criteria and incomplete device and stimulation description or monitoring [5, 6, 8]. Even though various bone growth stimulators are FDA-approved for clinical use [7, 9], after 60 years of research, ES devices for slow-healing or complicated fractures are only occasionally used in the USA and Canada [10]. We focused on direct contact stimulation with the background of finding safe and effective stimulation parameters for electrically active bone implants.

In general, the effects and mechanisms of ES at a molecular and cellular level are only partially understood. For investigating cellular effects of direct contact ES, direct current (DC) stimulation has been applied to mesenchymal stem cells or pre-osteoblasts in vitro. Pre-osteoblasts showed increased cell proliferation, enhanced surface attachment [11, 12] and expression of osteoblastic marker genes [13] upon stimulation. ES also influenced intracellular calcium ion (Ca^2+^) concentrations, as DC stimulation led to a 45-fold increase in Ca^2+^ levels in osteoblasts [14]. DC stimulation of mesenchymal stem cells prompted the cells towards enhanced osteogenic differentiation and osteogenic gene expression [15, 16]. Also, cells near the cathode exhibited changes in cell orientation, as their cytoskeleton aligned perpendicular to the electrical field [17]. Enhanced differentiation was accompanied by hyperpolarisation of the cell membrane [18].

The physical factor responsible for these effects is still discussed in the field of electrical bone tissue engineering. With direct ES, the cells are exposed to the electric field and the electrochemical products generated at the electrode interface. Direct DC stimulation can generate hydrogen peroxide (H_2_O_2_) at the cathode and change the pH value [19]. The electrochemical faradic by-products of DC stimulated medium were shown to affect cellular responses and might contribute to osteogenic differentiation but also cellular damage [20, 21]. In an attempt to reduce electrochemical reactions, experiments have been conducted using alternating current (AC) for direct contact ES. With the alternating current flow, electrochemical reactions can be reversed, as anode and cathode rotate. However, non-reversible faradic reactions can as well occur, when the applied charge exceeds the water window [22]. The AC stimulation parameters such as the waveform, frequency, duty cycle, voltage and current influence the induced charge and differ greatly in the literature [8].

AC stimulation with sinusoidal waveforms increased alkaline phosphatase activity [23], mineralisation and osteogenic gene expression [24]. Proliferation and vascular endothelial growth factor were found to be upregulated when biphasic currents were applied [25]. The application of 20 Hz biphasic, pulsed stimulation enhanced initial attachment and intracellular Ca^2+^ concentration [26]. Recent work suggests that AC stimulation also affects osteoclast viability and gene expression [27]. AC stimulation could therefore be beneficial for bone tissue engineering and facilitate enhanced osseointegration.

However, the effects of electrochemistry on the cells during the AC stimulation have not been studied so far. To differentiate the electrochemical and the electric field effects, we stimulated MG-63 directly or seeded the cells in previously stimulated cell culture medium. Previous studies from our group on physical plasma treatment showed that plasma treated cell culture medium induces the same cellular effects as the plasma treatment itself [28, 29]. We therefore hypothesized that medium, electrically stimulated with alternating currents, also contains electrochemical products such as H_2_O_2_, which induce the same effects as the direct ES. As intracellular reactive oxygen species (ROS) and Ca^2+^ play an essential role in cellular reactions to physical stimuli such as ES [30], this study examined these second messengers in connection with biphasic pulsed electrical stimulation. With a complete description and monitoring of our stimulation setup, other researchers can compare and recreate the effects of electrical stimulation.

## Materials and methods

### Electrical stimulation

**Fig. 1:**
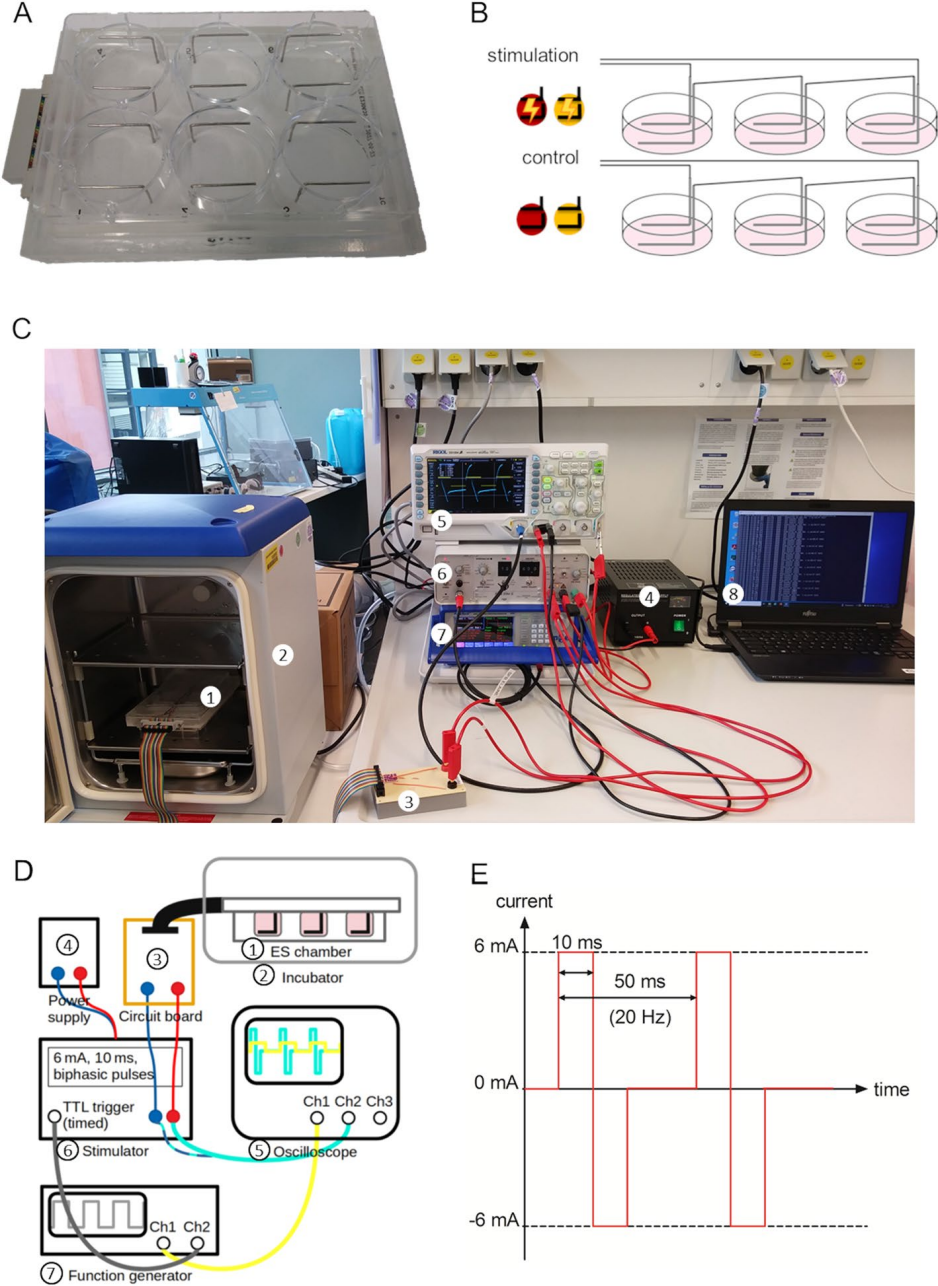
Electrical stimulation setup. **A** Bottom view of electrode lid (Mobini chamber) in a standard 6-well-plate. **B** Schematic view of Mobini chamber electrode setup and wiring. The symbols with lightning bolt indicate electrical stimulation of cells (red) or cell-free medium (yellow). In the parallel control approach no current was applied (symbols without lightning). **C** View of the stimulation setup with 1) Mobini chamber, 2) incubator, 3) circuit board, 4) power supply, 5) oscilloscope, 6) stimulator, 7) function generator and 8) laptop for data acquisition. **D** Schematic view of the ES setup and wiring. The function generator creates an “on-signal” at 20 Hz frequency. This is used as a trigger for the stimulator to produce the isolated biphasic 6 mA pulses. The resulting applied voltage across the three stimulated wells is measured with an oscilloscope (Ch1 = channel 1, Ch2 = channel 2, TTL trigger = timed transistor-transistor logic trigger). **E** Diagram of the applied current over time

For the electrical stimulation (ES), we used a self-build “Mobini chamber” (Fig. 1A). This chamber was first described for DC ES experiments by Mobini et al. [15]. The chamber fits on top of a standard 6-well-plate. L-shaped platinum electrodes are submerged in the culture medium. Three wells are stimulated in series (stimulation), while the other three wells containe electrodes without any applied electrical stimuli (control) (Fig. 1B). The stimulation setup consists of a stimulator (ISO-STIM-II, npi electronic GmbH, Tamm, Germany) powered by a DC power supply (VOLTCRAFT FSP-1132, Conrad Electronic SE, Hirschau, Germany) and driven by a function generator (GW Instek MFG-2230 M, dataTec AG, Reutlingen, Germany). The stimulation chamber was placed in an incubator at 37 °C, 5% CO_2_. Voltages were monitored using an oscilloscope (digital oscilloscope DS1054, Rigol Technologies Inc., Portland, USA) and the data were recorded on a computer (Fig. 1C,D). For AC stimulation, we applied current-controlled 10 ms rectangular biphasic pulses with 6 mA and 10 ms pulse width for 2 h at 20 Hz under standard culture conditions directly after cell seeding (Fig. 1E).

### Cyclic voltammetry

Electrochemical reactions at the platinum electrodes in cell culture medium were studied via cyclic voltammetry (CV) using a potentiostat (Autolab PGSTAT204, Metrohm AG, Herisau, Switzerland). To perform the CV characterisation, we fabricated a dummy device with two L-shaped Pt electrodes (wire of 0.6 mm diameter, 22 mm distance between electrodes) in a well of a 6-well plate. Therefore, all measurements correspond to a single well. The measurements were performed inside an incubator at standard cell culture conditions, 37 °C and 5% CO_2_. The three-electrode configuration was used. The working electrode and counter electrode were assigned arbitrarily to any of the electrodes of the stimulation device, as they are equivalent. An Ag/AgCl electrode (RE-1B, Biologic, Seyssinet-Pariset, France) with 6 mm diameter was used as a reference electrode. 4 ml fresh DMEM (Dulbecco's Modified Eagle Medium) was loaded to the system as electrolyte for each measurement. CV tests were performed within the voltage window (− 0.6 V to 0.9 V), where no reactions associated with water hydrolysis have been observed. Moreover, additional CV measurements have been carried out within the extended window (− 1.6 V to 2 V) obtained from voltage measurements. In all cases, cycles were performed at a sweep rate of 0.1 V/s and 10 mV step width. Several cycles were run until the system was stable. To determine the CV range, voltages were measured every 100 μs during 100 cycles (5 s) during stimulation. These measurements were carried out in the two electrodes configuration, using the same dummy device employed in the CV characterisation (Additional files Fig. A1).

### Electric field simulation and electrochemical impedance spectroscopy

We closely followed the methods described by Zimmermann et al. for the simulation of the applied electric field [31]. Briefly, a calibrated equivalent circuit model comprising the impedance of the electrode-electrolyte interface and the cell culture medium impedance was used to predict the stimulation pulses and the voltage drop across the medium in three wells in series. The calibration data were obtained from electrochemical impedance spectroscopy (EIS) spectra. We conducted the EIS on a single well at different input voltages (10 mV_rms_ to 1 V_rms_). The impedance spectra were recorded using a Reference 620 potentiostat (GAMRY instruments, Warminster, PA, USA). The conductivity of the cell culture medium DMEM was measured with a handheld conductivity meter (SevenGo Duo SG23 with probe InLab 751-4 mm, Mettler Toledo, Gießen, Germany) and was measured to be 1.46 Sm^−1^ at 35.5 °C. The probe had been calibrated with conductivity standards in the same range beforehand. The electric field was computed by the finite element method (FEM) using NGSolve 6.2.2102 [32], which is an open-source library for higher-order FEM built on top of the mesh generator NETGEN [33]. The voltage drop across the medium was used to set the boundary conditions in the FEM model.

### Cell culture and AC stimulation

Human osteoblast-like cells MG-63 (CRL1427™, ATCC, Manassas, USA) were cultured in DMEM GlutaMAX (Gibco, Thermo Fisher Scientific) with 10% fetal bovine serum (FBS Superior; Sigma Life Science, Thermo Fisher Scientific) and 1% gentamycin (5 mg/ml, Ratiopharm, Ulm, Germany). Cells were used from passages 5 to 30 [34]. For AC stimulation experiments, 20,000 cells/cm^2^ were seeded on glass coverslips in DMEM without pyruvate (Gibco, Thermo Fisher Scientific) with 10% FBS and 1% gentamycin and electrically stimulated (see above) for 2 h under standard culture conditions at 37 °C and 5% CO_2_ (ES cells). DMEM without pyruvate was used, as pyruvate is a scavenger of hydrogen peroxide [35] and we wanted to examine ROS.

To study medium-mediated effects, DMEM without pyruvate was stimulated as described above (ES medium) for 2 h. Cells were seeded in the ES medium shortly after stimulation and incubated for 2 h before further analysis. Cells were cultured up to 24 h after stimulation. To study long lasting effects of ES medium, the stimulated medium was stored at 37 °C, 5% CO_2_ for 7 days before cell seeding. For assessment of metabolic activity, cell amount, antioxidant concentration and calcium ion imaging additional experiments were conducted with 1 mM pyruvate (Sigma-Aldrich, Merck KGaA, Darmstadt, Germany) in the medium. As a control, unstimulated medium was supplemented with H_2_O_2_ (Fluka Analytical, Thermo Fischer Scientific) to a concentration of 10 μM. After ES the electrodes of the “Mobini chamber” were subsequently washed with A. dist., isopropanol (Walter-CMP GmbH & Co. KG, Kiel, Germany) and sterilised in UV light for at least 10 min each.

### pH-value and temperature

To assess the impact of ES on DMEM (without pyruvate), the pH value was measured after 2 h ES. Medium containing the electrodes (without ES) served as controls. The pH meter and pH probe with sensor tip (SI series, Sentron Europe BV, Leek, Netherlands) were used outside the incubator. The temperature of the culture medium was measured using an infrared camera (ThermaCAM™, FLIR Systems AB, Danderyd, Sweden). The camera was placed inside the incubator underneath the culture plate. Measurement points were positioned in the stimulation and control samples near the electrodes. Images of the bottom of the wells were taken every 5 min during treatment.

### Scanning electron microscopy and energy dispersive X-ray

Cells and medium were electrically stimulated as described above. Directly after stimulation or 2 h incubation in ES medium, cells were washed in phosphate buffered saline (PBS, Sigma-Aldrich, Merck KGaA, Darmstadt, Germany) and fixed with 2.5% diluted glutardialdehyde solution (Sigma-Aldrich, Merck KGaA). Fixed samples were washed with sodium-phosphate buffer (0.1 M), dehydrated in an ascending series of acetone and critical point dried (Emitech K850, Quorum Technologies LTD, East Sussex, UK). Samples were mounted on Al-SEM-carrier with adhesive conductive carbon tape (PLANO GmbH, Wetzlar, Germany) and coated with carbon (> 20 nm, CCU-010 HV, safematic GmbH, Zizers, Switzerland) or with gold (20 nm, EM SCD 004, BALTEC, Balzers, Liechtenstein) to reduce accumulation of electrostatic charge. Samples were analysed by a field emission scanning electron microscope (FE-SEM, MERLIN® VP Compact, Carl Zeiss AG, Oberkochen, Germany). Scanning electron microscopy images were taken from the selected regions with accelerating voltage 5 kV, working distance 5.1–5.5 mm. To image the electrodes, the “Mobini chamber” was mounted on a SEM-carrier with adhesive conductive Al-tape (PLANO GmbH). SEM-images were taken from the selected regions. Representative areas or interesting areas of the samples were analyzed for elemental composition with an energy dispersive X-ray detector (XFlash 6/30, Co. Bruker Corporation, USA) by the QUANTAX ESPRIT Microanalysis software (version 2.0). The applied detector and magnification are given in the explanations of the figures.

### Cell count and viability

Metabolic activity of adherent cells 24 h after ES treatment was assessed using CellTiter 96® AQ_ueous_ One Solution Cell Proliferation Assay (MTS; Promega GmbH, Walldorf, Germany). The tetrazolium compound is reduced by NADPH dependent dehydrogenases into a coloured formazan product. The amount of coloured product is therefore dependent on the metabolic activity of viable cells. To compare whether the metabolic activity of the viable cells was changed after ES, the amount of adherent cells was determined with crystal violet staining and used to normalise MTS absorbance.

As described before, 20,000 cells/cm^2^ were AC stimulated for 2 h and incubated at 37 °C, 5% CO_2_ for 24 h. Subsequently, non-adherent cells were acquired and cell count and viability were assessed using the NucleoCounter® NC3000™ with the Via1 cassette and Viability and Cell Count Assay (ChemoMetec, Allerod, Denmark). Cells were acquired and stained automatically with DNA binding acridine orange and 4′,6-diamidino-2-phenylindole (DAPI). Acridine orange is cell permeable and used to count all cells, while DAPI, which binds to the minor grove of adenine-thymine-rich regions of DNA [36], cannot diffuse across intact cell membranes and is used to stain and count dead cells in this assay. Adherent cells were incubated in MTS assay reagent diluted 1:6 in medium without pyruvate for 2 h. Absorbance was measured at 492 nm with a reference wavelength of 620 nm with a plate reader (anthos Mikrosysteme GmbH, Friesoythe, Germany). Afterwards, the cells were fixated with methanol (Sigma-Aldrich, Merck KGaA) for 10 min and stained with crystal violet (Neisser solution II, Carl Roth GmbH + Co. KG, Karlsruhe, Germany) for 15 min. Cells were washed with distilled water and the staining was extracted using 33% acetic acid (JT Baker, Avantor, Radnor, PA, USA). The absorbance was measured at 620 nm with the plate reader (anthos Mikrosysteme GmbH).

### Reactive oxygen species (ROS)

After 2 h ES, H_2_O_2_ was quantified in DMEM without pyruvate using the fluorimetric hydrogen peroxide assay kit (Sigma-Aldrich, Merck KGaA) according to manufacturer’s protocol. H_2_O_2_ is thereby detected via the reaction with a red peroxidase substrate through horseradish peroxidase and the generation of a fluorescent product. Fluorescence was measured with the fluorescence reader (ex/em: 540/590 nm) (infiniteM200, Tecan Group Ltd., Männedorf, Switzerland) before and after stimulation and H_2_O_2_ concentration was calculated from standard curve.

Intracellular ROS were detected using the 20,7′-dichlorofluorescein diacetate (DCFDA) assay (Abcam, Cambridge, UK) according to manufacturer’s protocol. DCFDA is a membrane-permeable compound that can enter the cells. Inside, cytoplasmic esterases deacetylate the compound into the non-fluorescent DCF. The dye is then oxidised by intracellular ROS, which makes it highly fluorescent. For our experiments, 1 × 10^6^ cells/ml were stained in suspension with 20 μM DCFDA for 30 min in the dark at 37 °C. Afterwards, cells were seeded in DMEM without pyruvate and then directly AC stimulated for 2 h. Fluorescence intensity was measured at 485 nm with a fluorescence reader (infiniteM200) before and after stimulation. The fluorescence of non-adherent cells was measured using flow cytometry (FACSCalibur, BD Biosciences, Ann Arbor, MI, USA). To differentiate ROS signal locations, adherent cells were stained after ES with 5 μM CellROX green (ROS in nucleus and mitochondria), 5 μM CellROX orange (ROS in cytoplasm) (Invitrogen GmbH, Darmstadt, Germany) and 9 μM Hoechst (Life Technologies, Thermo Fisher Scientific) for 30 min at 37 °C in the dark. 180 μM H_2_O_2_ served as positive control. After incubation the samples were washed with PBS and covered with medium without pyruvate. Images were acquired at random positions in the well and 10 cells per image were analysed using the Zen blue software (LSM 780, 40x/1 W Plan-Apochromat water objectiv, Carl Zeiss Microscopy GmbH, Jena, Germany; Software acquisition: Zen black 2.1 V 11.0.0.190; Software analysis: Zen 2.3 blue edition, Carl Zeiss Microscopy).

### Antioxidant assay

Intracellular ROS levels are tightly regulated by antioxidants such as ascorbate, glutathione and catalase [37]. In cell culture medium, 1 mM pyruvate is often added, which serves as ROS scavenger. Unspecific antioxidant concentration in cells was measured using the antioxidant assay kit (Cayman chemical, Ann Arbor, MI, USA). In short, the combined antioxidant ability of the sample to suppress the oxidation of ABTS (2,2-azino-bis(3-ethylbenzothiazoline-6-sulfonic acid) by metmyoglobin is compared to a Trolox standard curve.

Stimulated cells were collected in cold assay buffer (Cayman chemical) using a cell scraper. The cells were homogenised by sonication at 35 kHz (Sonorex RK 100, Bandelin electronic, Berlin, Germany) for 5 min. Samples were then centrifuged at 10,000 x g at 4 °C for 15 min (Eppendorf centrifuge 5417R, Eppendorf AG, Hamburg, Germany). Subsequently, the supernatant was collected and stored at − 80 °C. For the assay, samples were thawed and pipetted into a 96-well-plate. ABTS and metmyoglobin were added and the reaction was started by the addition of H_2_O_2_. Samples were incubated for 5 min and absorbance was read at 750 nm with a fluorescence reader (infiniteM200, Tecan Group Ltd., Männedorf, Switzerland).

### Aquaporins (AQP)

For general AQP expression, cells were cultured in DMEM with 1 mM pyruvate and subsequently processed. MG-63 cells were washed with PBS, trypsinised, and centrifuged (Centrifuge 5810 R, Eppendorf AG) for 5 min at 200 x g. The cells were then fixed with 4% PFA (in PBS, Sigma-Aldrich) at room temperature (RT). Cells were permeabilised with 0.1% Triton X-100 (Sigma-Aldrich) for 10 min at RT. After washing, incubation with 2% FBS (FBS Superior; Sigma Life Science, Thermo Fisher Scientific) in PBS was performed for 1 h to prevent non-specific binding of the primary antibodies. Subsequently, cells were washed and incubated with the primary antibodies at the given dilutions overnight at 4 °C (Table 1). The following day, cells were washed and incubated with secondary antibodies for 30 min at RT. Excess antibody was removed and cells were resuspended in 400 μl PBS and stored at 4 °C until analysis. Flow cytometry was performed using the FACSCalibur and Cellquest Pro software (4.0.1). FlowJo software (10.4.2; BD Biosciences) was used for analysis.

**Table 1:** Antibodies for aquaporin staining

Antibody	Lot	Supplier	Dilution
Aquaporin 1 Polyclonal Antibody	000021263	Invitrogen GmbH, Darmstadt, Germany	1:25 in PBS
Aquaporin 3 Polyclonal Antibody	B117761	Invitrogen GmbH, Darmstadt, Germany	1:25 in PBS
Aquaporin 5 Polyclonal Antibody	BJ02243284	Bioss Antibodies Inc., Woburn, MA, USA	1:50 in PBS (flow cytometry) 1:100 in PBS (microscopy)
Aquaporin 7 Polyclonal Antibody	BA10152932	Bioss Antibodies Inc., Woburn, MA, USA	1:50 in PBS (flow cytometry) 1:100 on PBS (microscopy)
Aquaporin 8 Polyclonal Antibody	Y03862639B	Invitrogen GmbH, Darmstadt, Germany	1:50 in PBS (flow cytometry) 1:100 in PBS (microscopy)
Aquaporin 9 Polyclonal Antibody	AG10277900	Bioss Antibodies Inc., Woburn, MA, USA	1:50 in PBS (flow cytometry) 1:100 in PBS (microscopy)
Aquaporin 10 Polyclonal Antibody	R93689	Invitrogen GmbH, Darmstadt, Germany	1:25 in PBS
Anti-Rabbit IgG Secondary Antibody Alexa Fluor 488	1678787	Life Technologies, Carlsbad, CA, USA	1:500 in PBS

Images of AQP 1 were acquired after fixation with ice-cold methanol (99.9%, Sigma Aldrich) for 10 min at 4 °C and permeabilised with Triton X-100 for 10 min at RT. Incubation with FBS (2% in PBS) was performed for 1 h, followed by addition of primary AQP 1 antibody (Table 1) and incubating overnight in a wet chamber at 4 °C. The samples were washed again with PBS and secondary antibodies were added for 30 min at RT. Fluoroshield mounting medium with DAPI (Fluoroshield™, Sigma-Aldrich) was added to the bottom of the cell container and covered with a coverslip (Menzel GmbH, Braunschweig, Germany). Samples were then stored at 4 °C until analysis with the LSM 780 confocal laser scanning microscope (Carl Zeiss). Images were taken with the Plan-Apochromat 63x oil immersion objective.

### Intracellular calcium ion (Ca^2+^) imaging

**Fig. 2:**
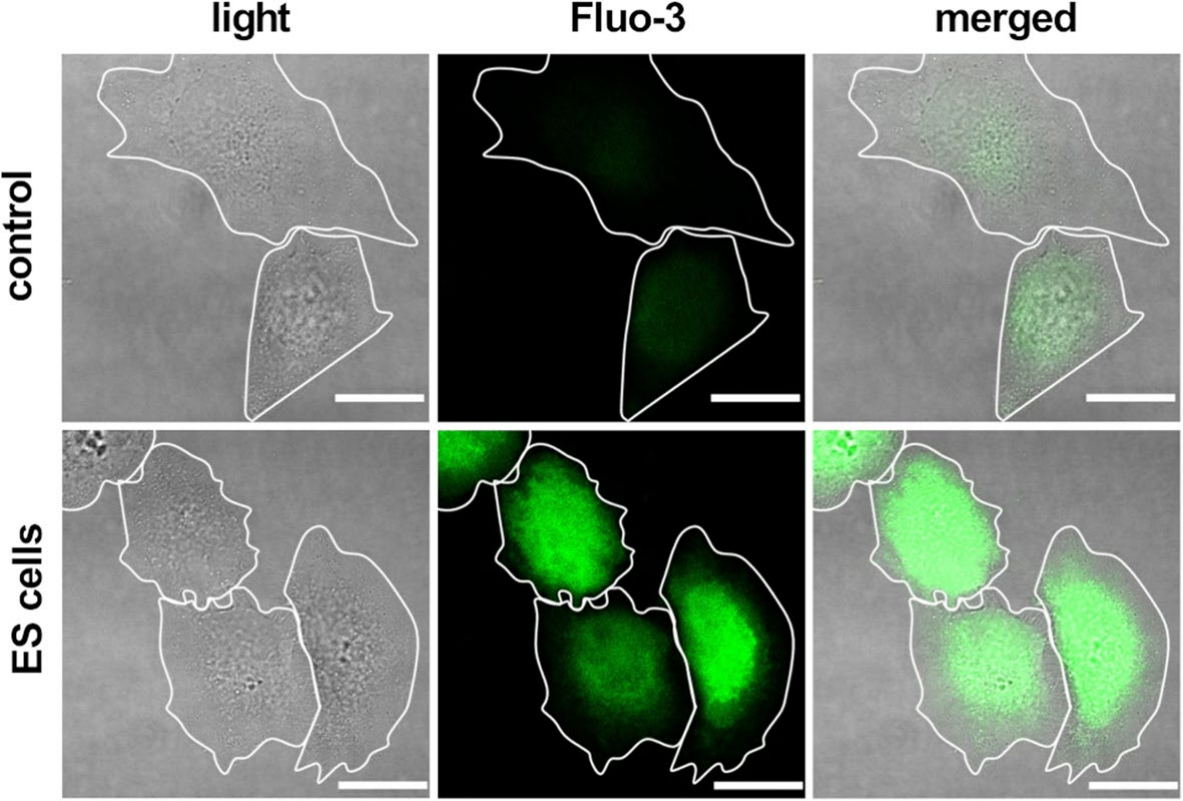
Image analysis of intracellular Ca^2+^: Mean fluorescence intensity (MFI) of MG-63 osteoblasts stained with Ca^2+^ sensitive dye Fluo-3. Cell shape was determined using light microscopy images from LSM. Then, MFI of Fluo-3 was measured over the whole cell area. (LSM 780, Carl Zeiss, scale bars 20 μm)

To evaluate the effects of AC stimulation on MG-63 s’ intracellular Ca^2+^ levels, 20,000 cells/cm^2^ were seeded in 6-well glass bottom plates (Cellvis, Mountain View, CA, USA). Cells were stimulated for 2 h directly after seeding as described before. After the stimulation, adherent cells were stained with 5 μM Ca^2+^ sensitive dye Fluo-3 acetoxymethyl ester (AM) (Life Technologies, Thermo Fisher Scientific) for 30 min in HEPES (4-(2-hydroxyethyl)-1-piperazineethanesulfonic acid) buffer (hypotonic and isotonic 1:1, Table 2) [38]. Afterwards, cells were resuspended in isotonic HEPES buffer. For assessment of ES medium on cellular Ca^2+^ levels, DMEM without pyruvate alone was stimulated for 2 h. MG-63 cells were stained with 5 μM Fluo-3/AM in HEPES buffer and seeded in ES medium. Fluorescence images were acquired using a confocal laser scanning microscope (LSM 780, Carl Zeiss Microscopy) with a Plan-Apochromat 63x oil immersion objective (Carl Zeiss Microscopy) and the Zen software (Zen 2.3 SP1 FP3 black, Carl Zeiss Microscopy). Fluorescence intensity was measured using the Zen software (Zen 2.3 blue edition, Carl Zeiss Microscopy). In well-spread cells the Fluo-3 dye and thus the calcium signal is spread over a greater area than in spherical cells. Therefore, bright field images of the LSM were used to determine the cell shape and area. Mean fluorescence intensity (MFI) of Fluo-3 was measured over the whole cell area (Fig. 2).

**Table 2:** HEPES buffer composition

component		isotonic HEPES	hypotonic HEPES
NaCl	[mM]	137	75
HEPES	[mM]	10	10
KCl	[mM]	5	2.75
D-Glucose	[mM]	5	5
Na_2_HPO_4_	[mM]	1	1
CaCl_2_	[mM]	1	1
MgCl_2_	[mM]	0.5	0.5
Bovine Serum Albumin	[g/L]	1	1

### Statistical analysis

Statistical analysis was performed using Graph Pad Prism 7.02 (GraphPad Software Inc., USA). The graphs show mean values with the standard error of mean (s.e.m). Data was tested for Gaussian distributions using the Shapiro-Wilk normality test. For comparison of multiple samples One-way ANOVA was used and for data, grouped by two or more factors, the two-way ANOVA was applied, both with Bonferroni post-hoc-tests. Where paired data was available for all data points, paired tests were used (RM-ANOVA). Wilcoxon matched-pairs signed rank test was used to test multiple, non-parametric, paired samples. Statistical tests are described in the figure captions. Significance levels are depicted as *p ≤ 0.05, **p ≤ 0.01, ***p ≤ 0.001 and ****p ≤ 0.0001.

## Results

### Characterisation of the ES chamber and AC stimulation

**Fig. 3:**
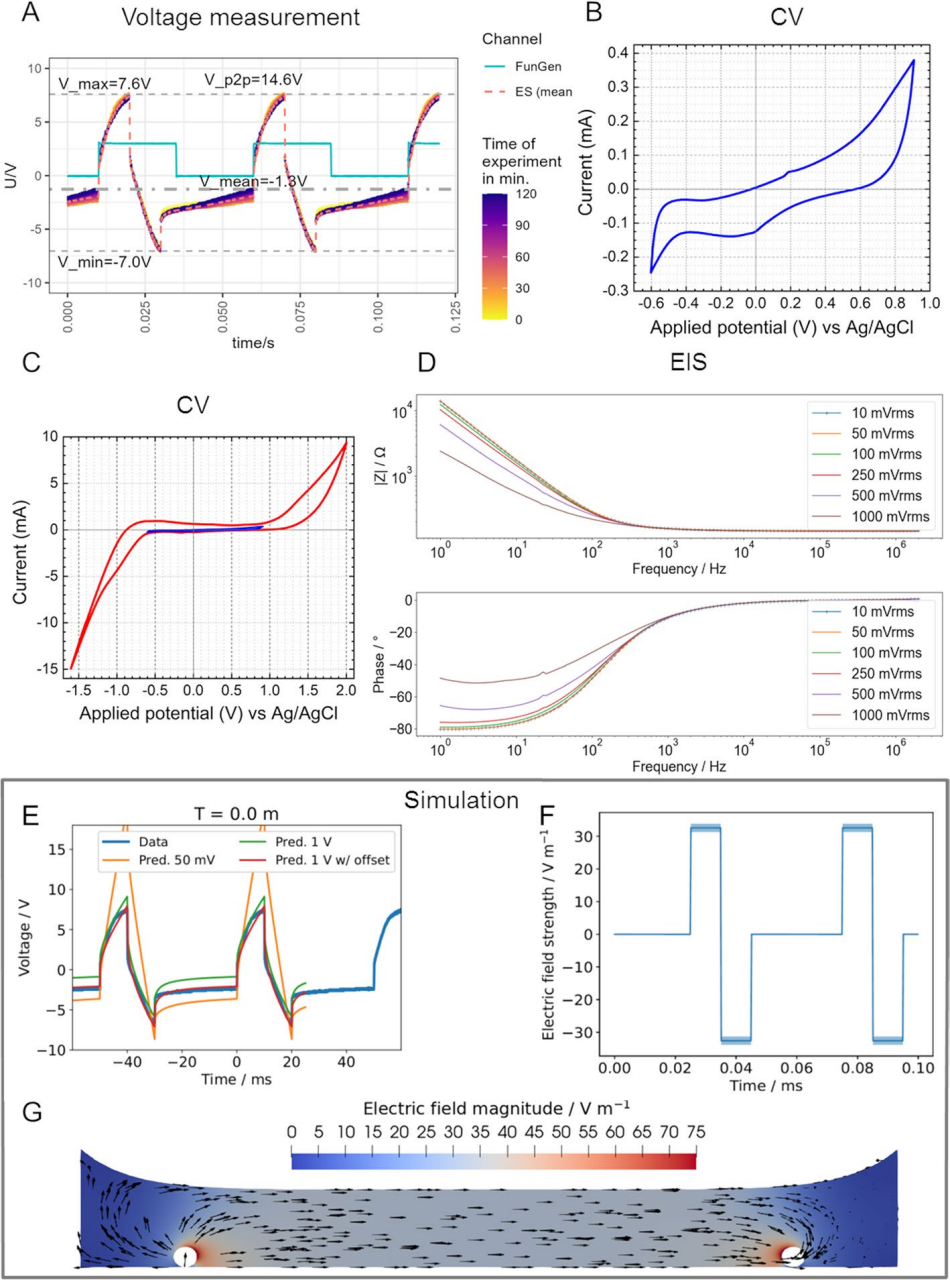
Characterisation of the electrodes and electric field during electrical stimulation (ES). **A** Voltage measurements over the course of the 2 ES. The colour code indicates the voltage over time. FunGen = 20 Hz frequency provided by the function generator. **B** Cyclic voltammetry (CV) was performed with a range from − 0.6 V to + 0.9 V. **C** Cyclic voltammogram within the voltage range of − 0.6 V to + 0.9 V (blue) compared to the extended range from − 1.6 V to + 2 V (red). Note that high currents appear at extended range. **D** Bode plot of electrochemical impedance spectroscopy (EIS). EIS was performed with input voltages from 10 mV_rms_ to 1 V_rms_. Note that, with input voltages of 0.5 and 1 V_rms_, the measured impedance (│Z│) changes. **E** Prediction of voltage flow from impedance measurements with 50 mV (orange), 1 V (green), 1 V and an arbitrary offset to match the data (red) in comparison to measured voltage flow during experiments (blue). Note that the simulation predicted the waveforms well, when EIS data with 1 V_rms_ input voltage was used. **F** Time-dependent simulated electric field strength in the centre of the well. **G** Simulated electric field distribution in the well. Note that the field is homogeneous except in close proximity to the electrodes

The application of 6 mA current pulses led to the resulting voltage of 14.6 V_peak to peak_ over the three wells in series (Fig. 3A). By dividing the overall voltage by 3, the peak to peak voltages per well amount to 4.87 V_peak to peak_. A negative voltage was measured in between the pulses, eventhough no current was applied. Over the course of the 2 h stimulation, this offset decreased. The applied voltage corresponds to the available driving force for electrochemical reactions.

For the electrochemical characterisation of the platinum electrodes, CV was performed. In the normally used range of − 0.6 to 1 V, the platinum oxidation and reduction can be seen as an increase in current at 0.2 and − 0.1 V respectively (Fig. 3B). We performed the CV also with an extended voltage range in regard to the measured voltages (Fig. 3C). The obtained cyclic voltammogram shows that the voltages attained during ES led to a vast increase in current. We concluded that the voltage window exceeded the water window and electrolysis took place at these points, producing chlorine and hydrogen at the negative potential and oxygen at the positive potential.

Furthermore, we performed EIS measurements for the calibration of the computational model. We found that the impedance measured with a high amplitude of + 1 V_rms_ led to a different impedance and phase at frequencies under 1 kHz than impedance measured with 10 or 100 V_rms_ (Fig. 3D). This change has an influence on the required voltage. Due to the high input amplitude, non-linear electrochemical effects could be observed. Based on the equivalent circuit model, we predicted the input voltage waveform to see if our model agrees with the experiment. The measured impedance could be successfully described by the equivalent circuit formulated by Zimmermann et al. [31]. However, the initially predicted input voltage from EIS measurements with 50 mV was far off from reality. The predicted waveforms based on EIS measurements with 1 V and the measured waveforms agreed relatively well (Fig. 3E). Additionally, in all stimulations the applied voltages were similar to each other (data available at 10.5281/zenodo.8262572).

As the medium is a pure resistor, the voltage drop across the medium should have the same shape as the applied current pulse. This is the reason why we chose current-controlled stimulation. Other than voltage-controlled stimulation, the voltage drop across the medium does in this case only depend on the resistance of the medium. Hence, we estimated the field using the conventional voltage-divider approach described in [31]. The magnitude of the electric field has been estimated to be between 31.3 and 33.8 V/m in the centre of the well (Fig. 3F). The field distribution in the well is homogeneous except for regions in close proximity to the electrodes (Fig. 3G).

### Physical changes of cell culture medium

**Fig. 4:**
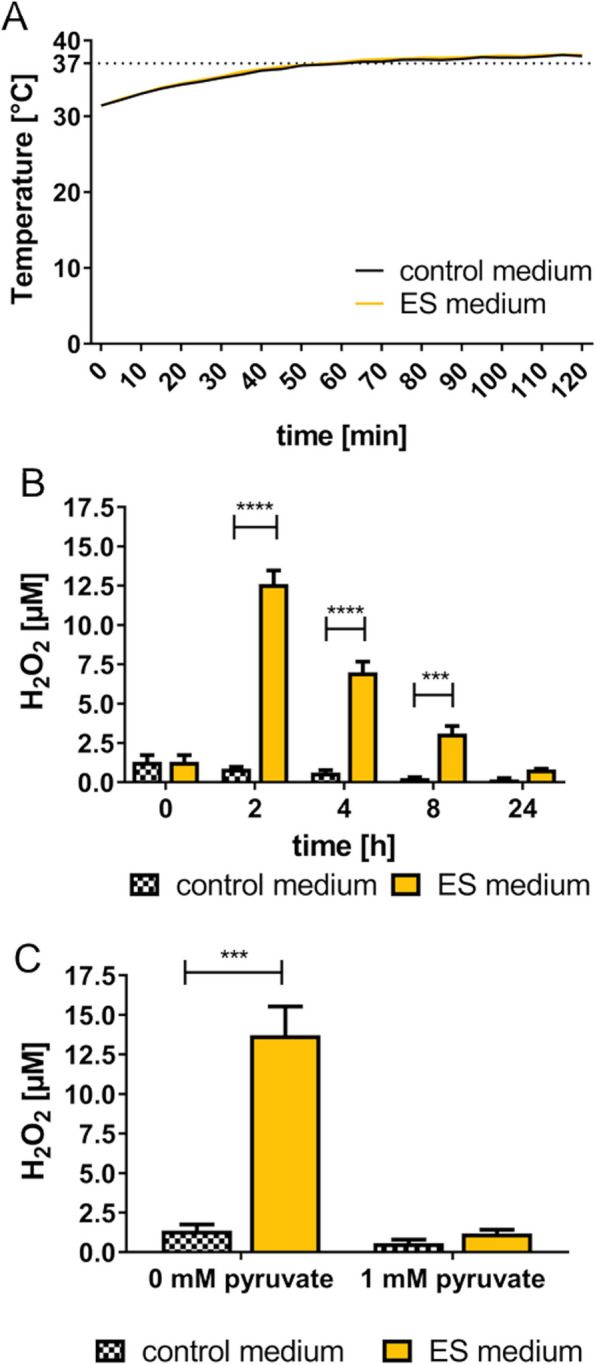
Changes in the medium induced by 2 h AC electrical stimulation (ES). **A** Temperature measurements during ES. Stimulation was performed inside the incubator with a target temperature of 37 °C (mean, *n* = 3 independent experiments). **B** Significantly higher H_2_O_2_ concentration in ES medium without pyruvate over the course of 8 h. Note the rapid decline of H_2_O_2_ up to 24 h (mean ± s.e.m., *n* = 2–3 independent experiments). **C** H_2_O_2_ concentration after 2 h ES in culture medium without and with 1 mM pyruvate added as ROS scavenger (mean ± s.e.m., *n* = 3 independent experiments). B and C: Two-way RM ANOVA with Bonferroni posttests, compared to control

The stimulation resulted in high voltage values up to almost 5 V_peak to peak_ per well. Therefore, as seen in the CV, water breakage is possible during stimulation. After 2 h ES of DMEM culture medium without pyruvate, we found no differences in temperature and pH-value between control and stimulated medium (Table 3). The temperature increased over the duration of the stimulation, as the samples were placed in an incubator with 37 °C as target temperature (Fig. 4A). We further investigated the H_2_O_2_ concentration before and up to 24 h after ES. The concentration significantly increased after 2 h of AC stimulation up to 12.6 ± 0.85 μM compared to 0.9 ± 0.11 μM in the control samples (electrodes placed in the medium). The concentration of H_2_O_2_ decreased in both groups over time. After 24 h there was no significant difference between the control (0.18 ± 0.08 μM) and the stimulation (0.83 ± 0.05 μM) (Fig. 4B). When 1 mM pyruvate was added, the H_2_O_2_ concentration did not increase after ES (Fig. 4C).

**Table 3:** Culture medium parameter after 2 h ES

	pH	Temperature [°C]	H_2_O_2_ [μM]
control	7.36 ± 0.02	37.97 ± 0.22	0.9 ± 0.11
ES medium	7.35 ± 0.02	38.17 ± 0.07	12.6 ± 0.85^****^

Mean ± s.e.m., *n* = 3 individual experiments, paired *t*-test

****p≤0.0001

### Morphology and adhesion

**Fig. 5:**
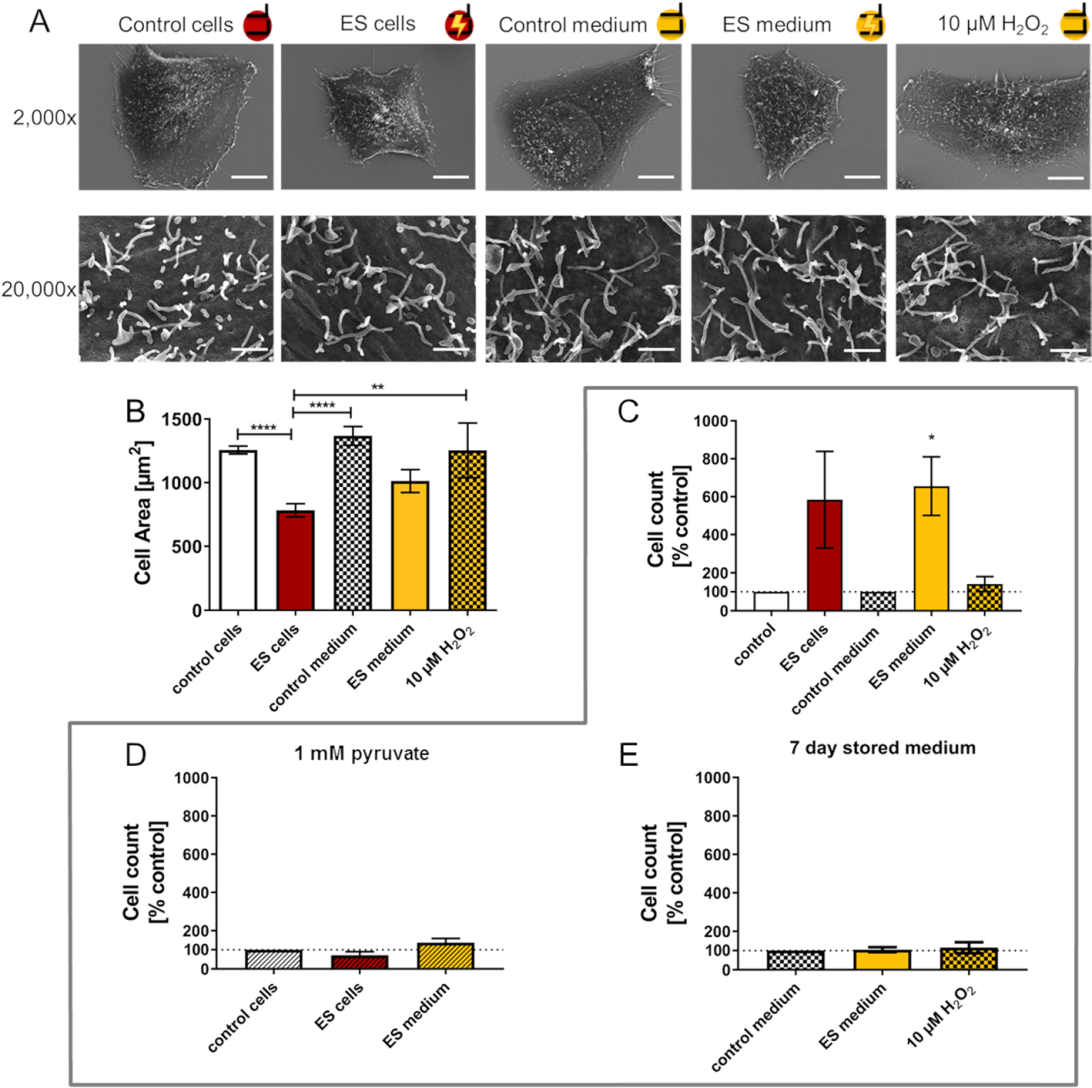
AC electrical stimulation (ES) influences adhesion of MG-63 cells. **A** Scanning electron microscopy images (SEM) of control cells, AC stimulated cells, cells in stimulated medium and cells in medium containing 10 μM H_2_O_2_ after 2 h of treatment. Upper row: adherent cells showing smaller cell area after ES (2000x magnification, detector HE-SE 2, scale bar 10 μm). Lower row: microvilli on the cell surface with no obvious changes in length and number (20,000x magnification, detector Inlens-Duo, scale bar 1 μm). **B** Cell area of adherent cells after 2 h ES (mean ± s.e.m., *n* = 4–7 independent experiments, One-way ANOVA with Bonferroni posttest). **C** Quantification of non-adherent cells 24 h after ES (mean ± s.e.m., *n* = 4 independent experiments, Wilcoxon matched-pairs signed rank test). **D** Quantification of non-adherent cells 24 h after ES with 1 mM pyruvate (mean ± s.e.m., *n* = 4 independent experiments, Wilcoxon matched-pairs signed rank test). **E** Medium mediated: quantification of non-adherent cells after 24 h in 7 days stored ES medium (mean ± s.e.m., *n* = 4 independent experiments, Wilcoxon matched-pairs signed rank test). Note that with this setup of 2 h AC both stimulation of cells and medium led to less adherent cells and more non-adherent cells. The addition of 1 mM pyruvate recovered the adhesion during treatment

For assessment of the cell morphology, SEM pictures of cells were taken after 2 h ES (Fig. 5A). The control cells on the cover glass were notably spread out. In contrast, the stimulated cells (ES cells) showed a rounder shape with fewer cells spread on the surface. Cells that were able to spread possessed long filopodia and microvilli. As we observed an increased H_2_O_2_ concentration in stimulated medium, we stimulated medium without cells and immediately seeded the MG-63 cells in ES or control medium. After 2 h the cells were fixated for scanning electron microscopy. ES medium also led to fewer cells spread on the cover glass as seen in ES cell samples. Other than expected, the addition of 10 μM H_2_O_2_ did not have the same effect on the cells as the ES medium. Cells were able to spread and adhere to the surface (Fig. 5A). The cell area was measured during Ca^2+^ imaging and ES cells were significantly smaller than the controls (Fig. 5B). Cells in ES medium also tended to be smaller than the cells in control medium or 10 μM H_2_O_2_ control.

Also, 24 h after stimulation, ES samples displayed more non-adherent cells, even though the result was not significant due to high variance (Fig. 5C). Cells incubated in ES medium showed significantly increased numbers of non-adherent cells. At the same time, addition of 10 μM H_2_O_2_ did not lead to this effect (Fig. 5C). On the other hand, if 1 mM pyruvate was added during the ES, the negative effects on the adhesion were recovered (Fig. 5D). If the stimulated medium without pyruvate was stored for 7 days before cell seeding, also no effect on cell adhesion was detected (Fig. 5E).

### Intracellular ROS production

**Fig. 6:**
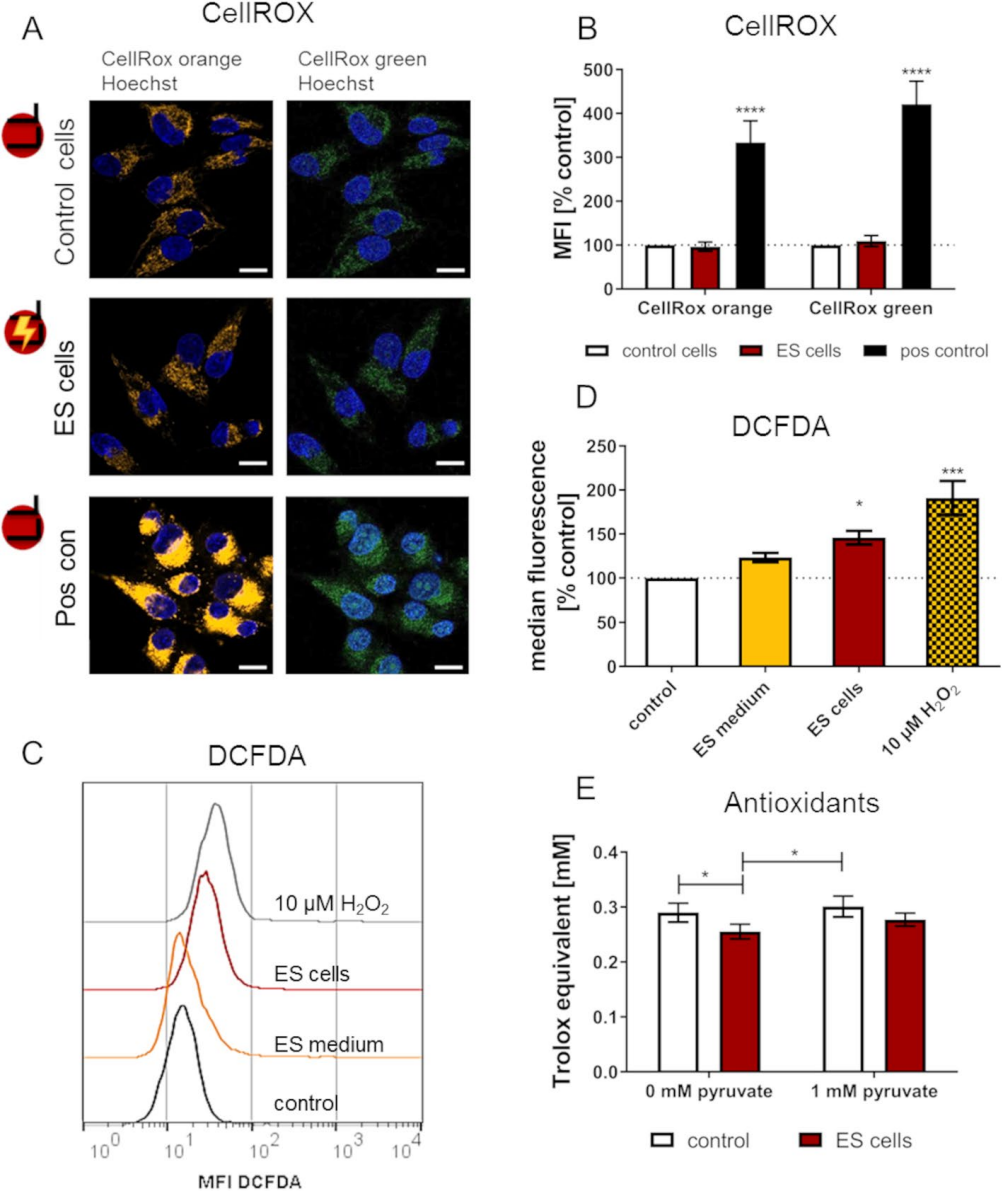
Electrical stimulation (ES) affects the redox system of MG-63 cells. **A** Fluorescence microscopy of intracellular ROS levels of adherent cells after 2 h ES. Note that ES does not influence intracellular ROS levels (LSM 780, scale bars 20 μm). **B** Mean Fluorescence Intensity (MFI) of ROS in the cytoplasm (CellROX orange) and ROS in the nucleus and mitochondria (CellROX green) (mean ± s.e.m., *n* = 5 independent experiments á 30–50 cells; Two-way ANOVA with Bonferroni posttests, compared to corresponding control). **C** DCFDA ROS fluorescence of non-adherent cells measured via flow cytometry (representative example of *n* = 3–4 independent experiments, flow cytometry). **D** DCFDA fluorescence (mean ± s.e.m., *n* = 3–4 independent experiments; One-way ANOVA with Bonferroni posttests, compared to control). E) Antioxidant concentration in ES cells stimulated in medium with and without 1 mM pyruvate (mean ± s.e.m., *n* = 3, Two-way ANOVA with Bonferroni posttests). Note that antioxidant concentration decreased in stimulated cells without pyruvate

As the AC stimulation led to increased H_2_O_2_ concentration in the medium and adversely affected the cell adhesion, we investigated the intracellular ROS response. We investigated directly stimulated, adherent cells using the CellROX staining for ROS in the cytoplasm (CellROX orange) and ROS in the nucleus and mitochondria (CellROX green). Fluorescence microscopy revealed no differences in ROS levels in ES cells compared to the control cells (Fig. 6A,B). The addition of 180 μM H_2_O_2_ as positive control increased ROS levels significantly, showing the possible maximum in cells’ ROS response. Subsequently we also investigated the ROS levels of non-adherent cells after 2 h direct and medium mediated ES. For this, cells were stained with the DCFDA assay and measured via flow cytometry (Fig. 6C,D). The ROS level of cells in ES medium did not significantly differ from the control. Directly stimulated ES cells showed an increase of 46 ± 7.7% compared to the corresponding control. Interestingly, the 10 μM H_2_O_2_-containing medium significantly increased the cellular ROS levels by 77.8 ± 19.9%.

### Antioxidation assay

The concentration of antioxidants in the cells was determined directly after ES. We stimulated the cells with and without 1 mM pyruvate and analysed adherent and non-adherent cells together. Cells stimulated in pyruvate-containing medium showed no difference in antioxidant concentration compared the unstimulated controls. The antioxidant concentration was 0.25 ± 0.02 mM in cells stimulated in medium without pyruvate and therefore significantly lower compared to the control’s concentration of 0.29 ± 0.02 mM without pyruvate and 0.30 (± 0.03) mM with pyruvate (Fig. 6E). The overall decline in antioxidant concentration is in accordance with the increased ROS levels of ES cell samples.

### Aquaporins (AQP)

**Fig. 7:**
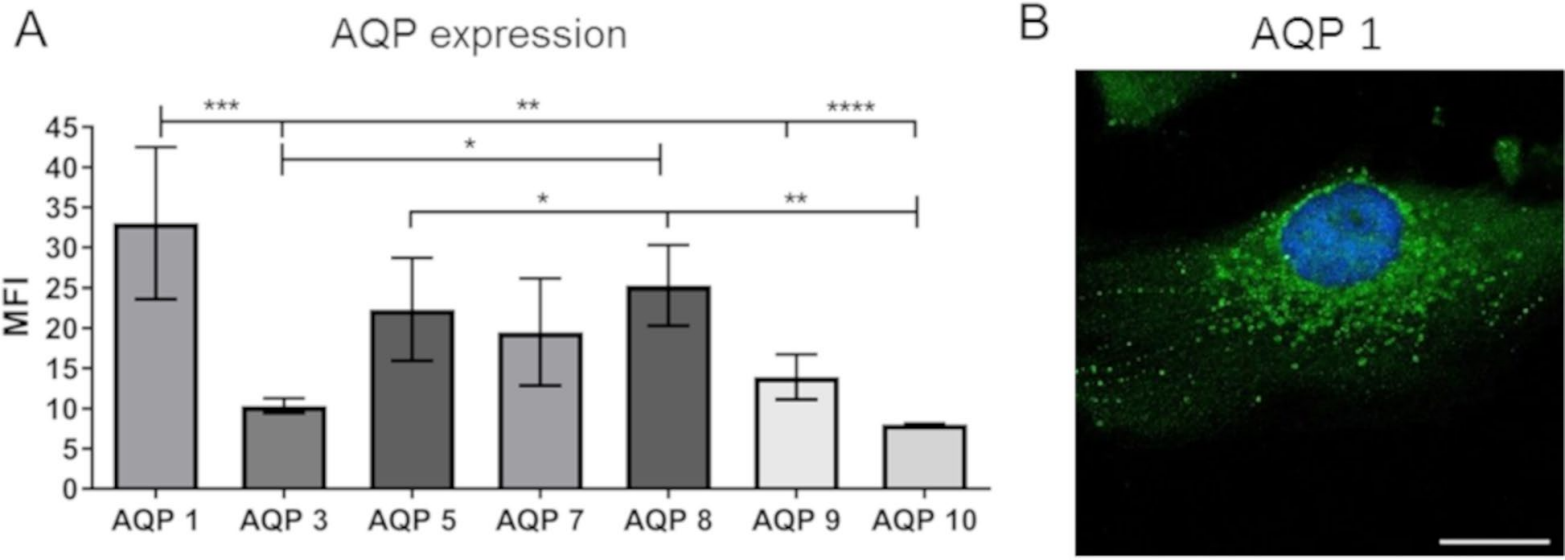
Aquaporin (AQP) channel expression in MG-63 osteoblasts. **A** Flow cytometry analyses of APQs. Note that AQP 1, as representative for H_2_O_2_ permeable water channel, is highly expressed (mean ± s.e.m., *n* = 3, One-way ANOVA with Bonferroni posttest). **B** Immunofluorescent staining of AQP 1 (green) and nucleus (blue, DAPI) (LSM 780, scale bar 20 μm)

AQPs are water channels of which some are able to transport H_2_O_2_ through the cell membrane [39]. H_2_O_2_ is an important by-product of the 2 h electrical stimulation system used here (see Fig. 4). Flow cytometry analysis of different AQPs in osteoblast-like MG-63 cells showed higher expression of AQP 1, 5 and 8 compared to AQP 3 and 10 (Fig. 7A). Immunofluorescent staining of AQP 1 showed a signal in the cytoplasm around the nucleus (Fig. 7B).

### Calcium ion signalling

**Fig. 8:**
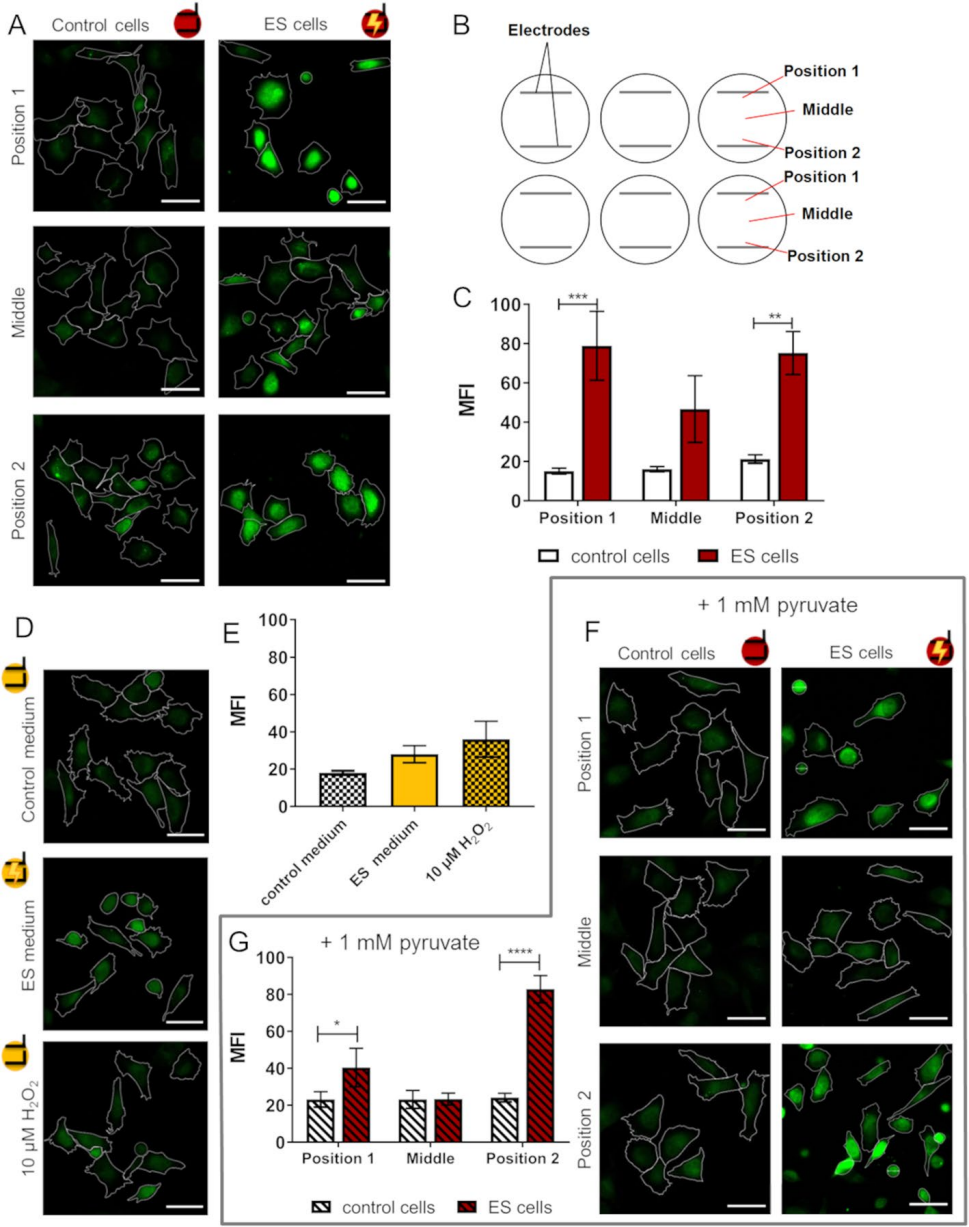
Intracellular calcium ion (Ca^2+^) levels are influenced by electrical stimulation (ES). **A** Fluo-3 stained cells directly after 2 h ES at different positions in the well. Note that Ca^2+^ levels are dependent on the position in the well. **B** Schematic view of microscopy positions in the well. Images were taken in the middle and close to the electrode positions. **C** Mean Ca^2+^ fluorescence at the different positions (mean ± s.e.m., *n* = 6–7 independent experiments, á 8–30 cells per position, Two-way ANOVA with Bonferroni’s multiple comparison test). **D** Intracellular Ca^2+^ of Fluo-3 stained cells seeded for 2 h in ES medium vs. medium with 10 μM H_2_O_2_. Note that Ca^2+^ levels are not influenced. **E** Mean Ca^2+^ fluorescence of medium mediated ES (mean ± s.e.m., *n* = 4, RM One-way ANOVA with Bonferroni’s multiple comparison tests, n.s.). **F** Fluo-3 stained cells after direct ES for 2 h in DMEM with pyruvate at different positions of the wells. **G** Mean Ca^2+^ fluorescence of ES cells in pyruvate-containing medium at different positions. Note that Ca^2+^ levels only slightly increase at position 1. (mean ± s.e.m., *n* = 6 independent experiments, á 14–38 cells per position, comparison within the positions with RM Two-way ANOVA with Bonferroni’s multiple comparison test). LSM 780, Carl Zeiss, scale bars = 50 μm

As intracellular Ca^2+^ ions are important second messengers, we investigated the Ca^2+^ levels of adherent cells after 2 h ES. Interestingly, the position in the well influenced the intracellular calcium signal of the ES cells (Fig. 8A). Images were taken in different regions of the well: in the middle of the well and at Positions 1 and 2, which were located near the electrodes (Fig. 8B). The Ca^2+^ levels where significantly elevated in ES cells in areas near the electrodes (Fig. 8C). Whereas in the middle of the well cells tended to have increased Ca^2+^ levels but the changes were not significant. This pattern follows the field strength distribution with around 30 V/m in the middle of the well and up to 65 V/m at the electrodes (Fig. 3E). Concerning medium mediated effects, after 2 h in ES medium, cells showed a trend towards increased Ca^2+^ levels compared to cells in control medium (Fig. 8D, E). This increase was not significant or as prominent as in ES cells. Adding 10 μM H_2_O_2_ also slightly increased Ca^2+^ levels but did not lead to prominent results as direct stimulation of cells. To investigate the impact of not only H_2_O_2_ but other ROS during the stimulation, the cells were also directly stimulated in medium supplemented with 1 mM pyruvate. At position 1 near the electrode, the rise in Ca^2+^ concentration through ES was decreased but still significant compared to the control. In the middle of the well no effects of the ES on Ca^2+^ levels were observed. At position 2 near the electrode, ES cells still showed a huge and significantly increased Ca^2+^ signal (Fig. 8F, G).

Taken together, direct stimulation and medium-mediated stimulation are not interchangeable stimulation methods (Table 4). While they both decreased cell adhesion and spreading, the intracellular messengers ROS and Ca^2+^ were increased after direct stimulation but not through medium-mediated treatment. Interestingly, the external addition of 10 μM H_2_O_2_ had no effect on the adhesion but significantly increased intracellular ROS.

**Table 4:** Summary of cellular effects after ES treatment

	ES cells	ES medium	10 μM H_2_O_2_
Adhesion	↓	↓	–
Intracellular ROS	↗	–	↑
Intracellular Ca^2+^ level	↑	–	–

## Discussion

### Characterisation of the AC stimulation parameters

A digital twin is a model of the stimulation chamber that is continuously updated by measurements. The digital twin of the stimulation chamber permits to estimate the applied electric field reliably and in accordance with continuously monitored stimulation parameters. An initial guess is formulated based on impedance spectroscopy. Zimmermann et al. already established a digital twin of the “Mobini chamber” [31]. However, the digital twin was only validated for stimulation parameters typical of neural stimulation (in particular, deep brain stimulation). We used the same approach and tested if it could be straight-forwardly applied to our parameters.

In contrast to previous results, we found that non-linear electrochemical effects play a significant role when using our stimulation parameters. These effects manifested themselves in the theoretical analysis of the waveforms based on impedance spectroscopy data. Furthermore, both an offset of the applied voltage and a minor waveform change with time could be observed. Merrill et al. described possible electrochemical processes taking place at the platinum electrodes during ES [22]. Aside from the capacitive charging of the electric double layer, reversible and irreversible faradaic reactions with charge transfer can occur, depending on the applied stimuli. Irreversible reactions happen when the counter pulse cannot immediately reverse an electrochemical reaction. Then, reaction products remain in the medium or are released from it. These irreversible reactions lead to a ratcheting of the applied voltage until the same amount of charge is lost during the anodic and cathodic pulse, leading to the observed voltage offset, also being called excess potential or residual voltage [22].

In the field of ES application for bone tissue engineering, many different stimulation systems and parameters are in use [8]. Authors usually report the electric field strength to compare the applied stimulation. However, the methods for obtaining the field strength often remain unnamed and the description of pivotal parameters such as current, voltage, conductivity of the medium and impedance measurements are incomplete [40]. Parameter studies linking specific voltages, frequencies and currents [12, 41, 42] to cellular effects cannot be compared, and the results cannot be applied to other systems. The same problem is seen in pre-clinical trials. A standard set of applied parameters should always be reported [6].

Using the digital twin, we have a reliable electric field estimator at hand. Moreover, we have shown that considerable electrochemical reactions take place that need to be accounted for in interpreting experimental results. We encourage other researchers to follow our example and provide detailed characterisation and monitoring data of their electrical stimulation experiments.

### Physical changes of cell culture medium and influence of H_2_O_2_

After AC stimulation with our parameters using the “Mobini chamber”, the temperature and pH value of the culture medium remained unchanged, while the H_2_O_2_ concentration was increased to about 12 μM. At high intracellular concentrations, ROS like H_2_O_2_ react with proteins, lipids, carbohydrates and nucleic acids and can thereby cause functional changes or the destruction of these biomolecules [43]. At moderate concentrations ROS enable redox-dependent biosynthetic processes and are part of signalling pathways [44, 45]. A 10 to 100-fold concentration gradient from extracellular to intracellular H_2_O_2_ concentration can be assumed, even though higher and lower gradients were described. An extracellular H_2_O_2_ concentration of 10 μM, as we observed, would therefore result in an intracellular concentration of 0.1 to 1 μM, which is still described to result in beneficial responses [46, 47]. Srirussamee et al. [20] also investigated their cell culture medium for physical changes after DC ES and, similar to our results, found the pH value unchanged and H_2_O_2_ concentration increased to 5 μM after 2 h stimulation [20]. They also treated the cells with the stimulated medium and described cellular effects like decreased metabolic activity. However, it has to be considered that the assay they used shows the amount of metabolically active cells. The measurements must be normalised to the number of cells in the sample to describe the metabolic activity. In our experiments, the cell numbers decreased significantly when treated with stimulated medium. When we normalised the metabolic activity to the cell amount, no difference was found between ES and control (Additional files Fig. A2).

Adding 10 μM H_2_O_2_ to our control medium had neither detrimental nor beneficial effects on cell adhesion. This leads to the conclusion that the H_2_O_2_ production through ES is not one of the main contributors to the cellular effects. On the other hand, the addition of 1 mM pyruvate to the cell culture medium inhibited the detrimental effects of ES. In another study, Srirussamee et al. [21] also applied 1 h stimulated media to mesenchymal stem cells and found an increase in metabolic activity. When they added 5 mM pyruvate to scavage ROS, the effect was inhibited [21]. Therefore, H_2_O_2_ and other ROS might play an important role in bone regeneration. However, radical oxygen or nitrogen species are difficult to detect, as they react with other substances, such as amino acids, ascorbic acid or folic acid [48–50]. Therefore, they are only present for some nano- to microseconds [51].

H_2_O_2_ cannot diffuse freely through cell membranes but requires channels. Aquaporins (AQP) are a family of transmembrane water channels regulating the transfer of water, gases and small solutes such as urea and glycerol [52]. Some AQPs are also able to transport H_2_O_2_ across membranes [39]. AQPs 1, 3, 5, 8 and 9 have been described in mammalian cells and human stem cells to be permeable for H_2_O_2_ [39, 52]. Our results indicate that MG-63 cells express AQP 1, 5, 7 and 8. Therefore, extracellular H_2_O_2_ may enter the cell, thereby increasing intracellular ROS levels and reducing antioxidant concentration. Surprisingly, although the ES treatment led to increased H_2_O_2_ concentrations in the medium, ROS levels of cells incubated in ES medium did not significantly increase. Cells incubated in the control medium with 10 μM H_2_O_2_ showed the largest increase in intracellular ROS levels.

We hypothesise that other factors may interfere in ES samples with the generated H_2_O_2_ or inhibit the transport through the AQPs. Using the “Mobini chamber”, Srirussamee et al. found enhanced platinum concentrations of 34 μg/L in the culture medium after their ES treatment [21]. On one hand, MG-63 cell culture exposed to platinum showed no growth inhibition or morphological changes [53]. Also, many medical products such as deep brain stimulation electrodes consist of platinum for its good biocompatibility [54]. On the other hand, the dissolution and corrosion of platinum electrodes during stimulation depend on charge density and pulse duration [55]. With our stimulation regime, irreversible reactions at the electrodes are likely to include corrosion of the platinum with chloride ions from the medium (Pt + 4Cl^−^ → [PtCl4]^2−^ + 2e^−^) [22, 55]. However, scanning electron microscopy images and energy dispersive X-ray analysis of the structural and elemental compositions did not show concrete evidence for platinum corrosion (Additional files Fig. A3). On the one hand, the electrodes, which served as controls, as no current was applied to them, showed a smooth surface with only a few scratches. On the other hand, some electrodes, which were used for the electrical treatment, showed apparent changes of the surface. However, this was only seen in 50% of the stimulated electrodes. As expected, the element analysis showed a high signal intensity for platinum in all samples. Traces of carbon and oxygen were found on some residues, which are likely organic debris. Traces of chloride were found but also sodium chloride crystals. In contrast, in preliminary experiments, platinum could be detected in culture medium after stimulation. Therefore, enhanced platinum concentrations in the medium may also contribute to the cellular effects. Martins et al. showed that metal compounds can inhibit AQP 1 and 3. The gold(III) based complexes were able to inhibit especially AQP 3 in red blood cells, however platinum-based compounds did not show significant effects [56, 57]. Inhibition of AQP 1 by silencing of the expression with shRNA led to decreased extracellular matrix adhesion, proliferation and cell survival in MG-63 cells [58]. Further experiments need to be conducted for a better understanding of possible cellular effects of platinum corrosion products.

Furthermore, we investigated whether stored media still mediates cellular effects after 7 days. For example, medium-mediated effects of physical argon plasma persist even after 7 and 21 days of storage [28, 29]. The authors hypothesised the formation of stable organic peroxides in cell culture medium by plasma-induced ROS chemistry [28]. We found no effects of stored ES medium on cells. Therefore, medium-mediated ES effects were not long-lasting and the DMEM did not preserve the electrochemical characteristics once induced by the electrical stimulation.

### Intracellular calcium ion levels

Ca^2+^ is an important intracellular second messenger and changes of intracellular Ca^2+^ concentrations are widely believed to contribute to cellular effects of ES in bone-forming cells [4, 30]. In DC stimulation experiments osteoblast-like cells showed rapidly increased intracellular Ca^2+^ levels upon start of the stimulation [59]. The application of 10 min biphasic stimulation also led to a direct increase of Ca^2+^ [26]. Even though the Ca^2+^ level increase did not persist over 2 days of ES, gene expression of Ca^2+^ signalling pathway-related genes and osteogenesis-related genes were upregulated [60]. Ca^2+^ levels increased up to 45-fold after 20 min of stimulation [14]. In some experiments, the rise of intracellular Ca^2+^ concentration was dependent on the influx of extracellular Ca^2+^ [59]. Other studies reported the increase to be independent of extracellular Ca^2+^ [26] or that the release of Ca^2+^ from intracellular stores plays a significant role in cytoplasmic Ca^2+^ increase [14].

MG-63 cells showed differences in intracellular Ca^2+^ levels in response to physical factors such as the biomaterial they were cultivated on. Material with moderately positive surface charges showed beneficial effects on cell viability, increased Ca^2+^ levels and enhanced Ca^2+^ mobilisation in MG-63 [61, 62]. Also, mechanical stimulation increased the concentration of intracellular Ca^2+^ via mechano-sensitive ion channels like Piezo1, which is highly important in bone physiology [30, 63]. Enhanced Ca^2+^ levels therefore pointed towards improved cell performances. In contrast, Ca^2+^ also plays a major role in programmed cell death and apoptosis [64]. Disruption of Ca^2+^ homeostasis can also lead to necrotic cell death [65]. ES has been described to induce or inhibit apoptosis depending on the ES parameters. In both cases, increased Ca^2+^ concentrations mediate downstream signalling cascades and the cells’ fate [66].

In our experiments, the Ca^2+^ level was found to be increased in regions near the electrodes. Cells in ES medium or H_2_O_2_ control did not show such a large increase. This effect was therefore not mediated by stable products in the medium and might be a reaction to the electric field. Near the electrodes, the electric field is higher than in the rest of the well and might influence the Ca^2+^ levels. However, when the ROS scavenger pyruvate was added to the culture medium during ES of cells, Ca^2+^ level increase was diminished only at one electrode. At the other electrode, this reduction was not seen. The reason for these position-dependent changes might be the pulsed stimulation with 30 ms stimulation breaks in each cycle. The electrode at position 2 is first the cathode and then the anode during the biphasic stimulation pulse. Cathodic reactions include the reduction of water. Anodic reactions include oxidation of water and corrosion of platinum with chloride ions [22]. While faradic products from the cathodic reaction can be reversed by the anodic pulse, the generated faradic by-products of the anodic phase are not directly reversed. Therefore, locally high concentrations of faradic by-products might also contribute to the increase in intracellular Ca^2+^ at one electrode despite the pyruvate. Intracellular Ca^2+^ and ROS pathways are connected and influence each other. For example, calcium channels in the cell membrane and endoplasmic reticulum are redox-sensitive and modulated by ROS to increase cytosolic Ca^2+^ influx [67, 68].

Switching the electrode connections to change which electrode is cathode first did not change the location of increased Ca^2+^ levels under ES with pyruvate. It seems likely that repeated usage of the “Mobini chamber” has changed the electrodes differently depending on the electrode being anode or cathode first. In the CV experiments, the generated current changed throughout multiple cycles (Additional files Fig. A4). This effect was described before and was attributed to oxidation of surface atoms, platinum dissolution and redeposition [69]. The electrochemical potential of each electrode is influenced based on anodic- or cathodic-first pulses [70]. For further biological studies, the platinum electrode surface state should be considered. The different potentials of the electrodes should be prevented by constantly changing the order of the electrodes in the circuit.

## Conclusion

We wanted to determine whether electrical stimulation effects on cells are mediated by the electric field or electrochemical products, applied by AC pre-conditioned medium. The direct and indirect, medium-mediated electrical stimulation of MG-63 cells led to decreased adherence of the cells after 2 h alternating current stimulation. When hydrogen peroxide (H_2_O_2_) was added in the same concentration as measured after electrical stimulation, this effect could not be observed. The cells showed significantly increased intracellular ROS levels when incubated with the 10 μM H_2_O_2_ control but not with ES medium and only slightly in directly stimulated cells. Neither electrically stimulated medium nor H_2_O_2_ increased the intracellular calcium levels like direct electrical stimulation of cells did. Therefore, the increased electric field at about 65 V/m at the electrodes might be responsible for the increased intracellular calcium ion concentration. However, as the addition of pyruvate diminished the calcium ion increase, ROS concentration gradients might as well play a part. Taken together, ES led to changes in the culture medium, including the generation of H_2_O_2_. Nevertheless, the electrical stimulation of the culture medium alone did not induce the same cellular effects as the direct stimulation of cells, indicating the impact of the electric field.

### Supplementary Information

**Additional file 1: Fig. A1**. Applied voltages measured during stimulation with 6 mA in a single well.

**Additional file 2: Fig. A2**. Metabolic activity of MG-63 24 h after electrical stimulation (ES) in medium with and without 1 mM pyruvate. A) Electrically stimulated cells. B) Cells incubated in stimulated medium. (Both: mean ± s.e.m., *n* = 3, Two-way RM ANOVA with Bonferroni posttests).

**Additional file 3: Fig. A3**. Scanning electron microscopy (SEM) and energy dispersive X-ray (EDX) analysis of the platinum electrodes. A) SEM images of representative electrodes from the “Mobini chamber”. Control = no current was applied, ES = electrical stimulation, electrodes were used to deliver 6 mA current pulses. B) EDX measurements for element analysis. C = carbon, Cl = chloride, O = oxygen, Pt = platinum.

**Additional file 4: Fig. A4**. Impact of electrode cycling on cyclic voltammetry (CV). CV was performed for up to 23 cycles. Note the change in current over the number of cycles.

## Data Availability

The datasets supporting the conclusions of this article are available in the zenodo repository 10.5281/zenodo.8262572.

## Abbreviations

AC: alternating current
AQP: aquaporin
Ca^2+^: calcium ions
CV: cyclic voltammetry
DC: direct current
EIS: electrochemical impedance spectroscopy
ES: electrical stimulation
H_2_O_2_: hydrogen peroxide
