# Supplementary figures and images for: Discrimination between the effects of pulsed electrical stimulation and electrochemically conditioned medium on human osteoblasts
Source: J Biol Eng. 2023 Nov 23;17:71. doi: 10.1186/s13036-023-00393-1 (PMC10668359; doi:10.1186/s13036-023-00393-1)

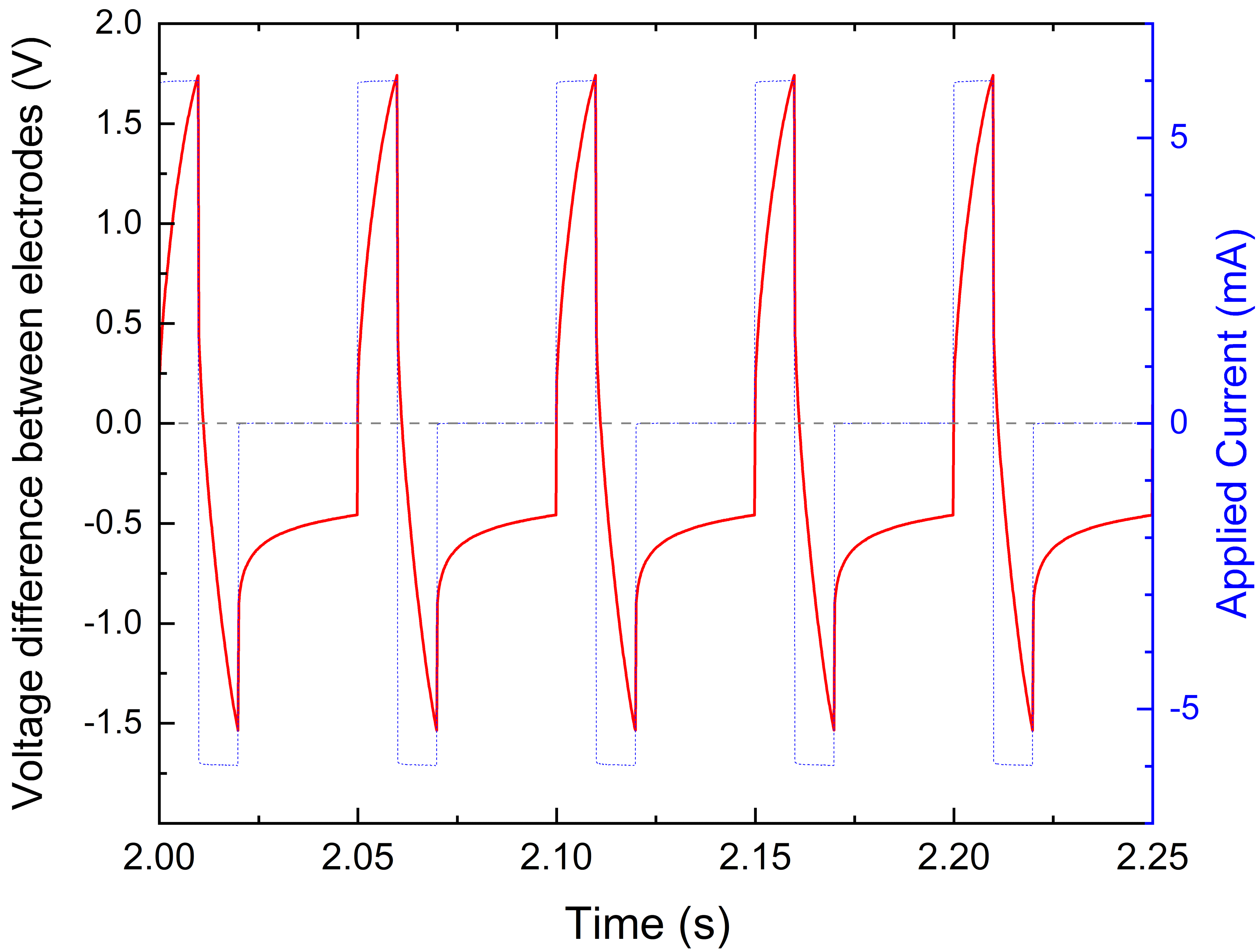

Supplement: Supplementary file 1 — Additional file 1: Fig. A1. Applied voltages measured during stimulation with 6 mA in a single well. [file 13036_2023_393_MOESM1_ESM.tif]

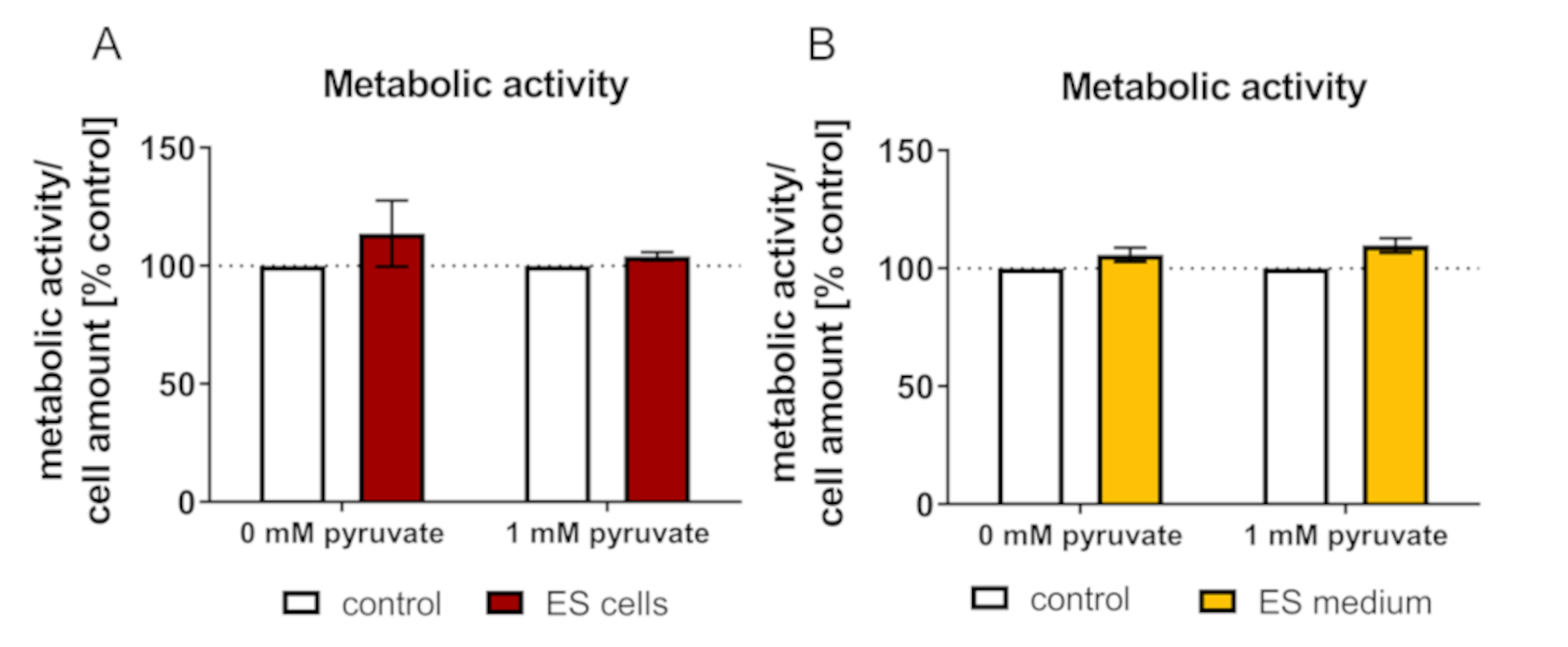

Supplement: Supplementary file 2 — Additional file 2: Fig. A2. Metabolic activity of MG-63 24 h after electrical stimulation (ES) in medium with and without 1 mM pyruvate. A) Electrically stimulated cells. B) Cells incubated in stimulated medium. (Both: mean ± s.e.m., n = 3, Two-way RM ANOVA with Bonferroni posttests). [file 13036_2023_393_MOESM2_ESM.tif]

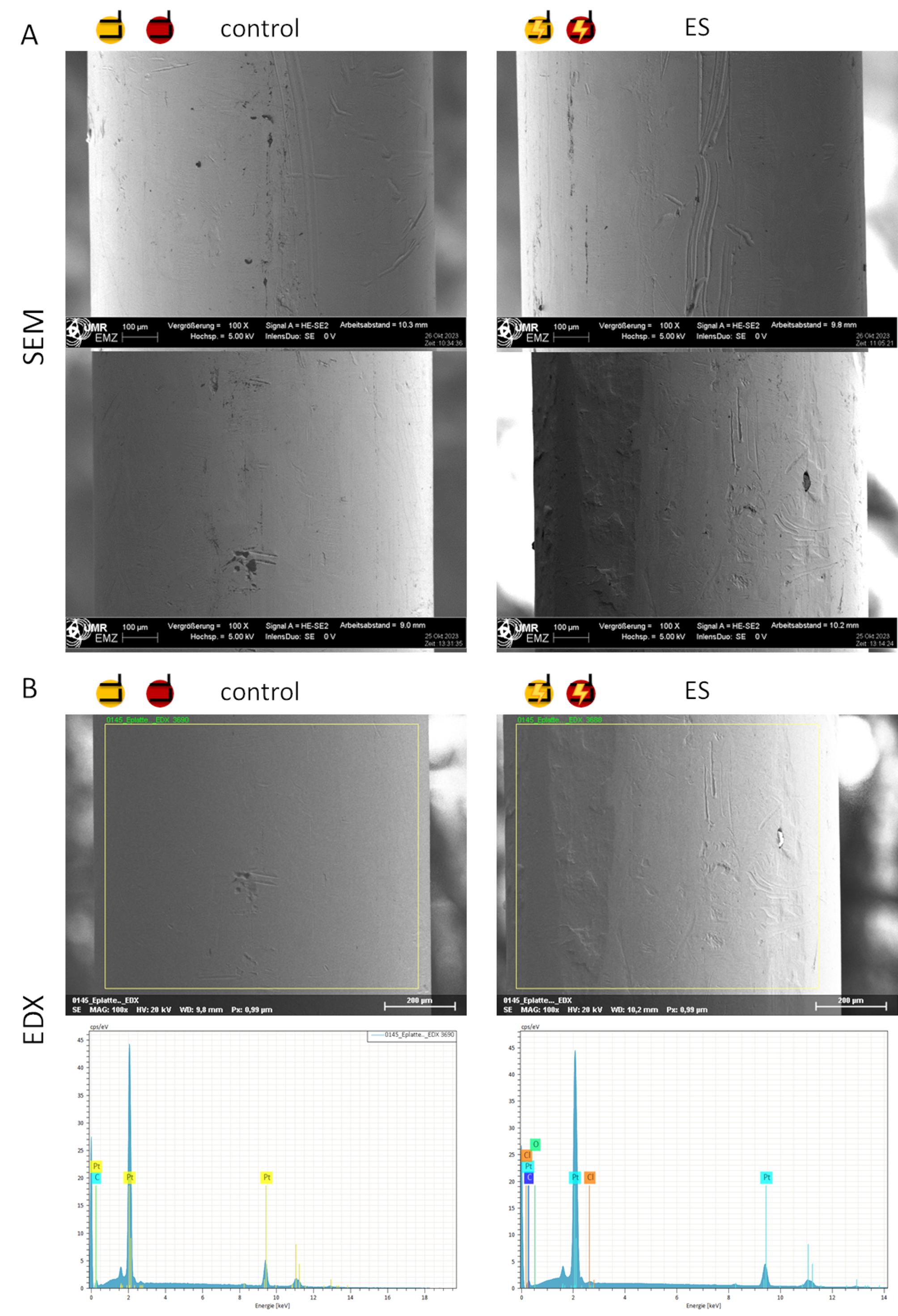

Supplement: Supplementary file 3 — Additional file 3: Fig. A3. Scanning electron microscopy (SEM) and energy dispersive X-ray (EDX) analysis of the platinum electrodes. A) SEM images of representative electrodes from the “Mobini chamber”. Control = no current was applied, ES = electrical stimulation, electrodes were used to deliver 6 mA current pulses. B) EDX measurements for element analysis. C = carbon, Cl = chloride, O = oxygen, Pt = platinum. [file 13036_2023_393_MOESM3_ESM.tif]

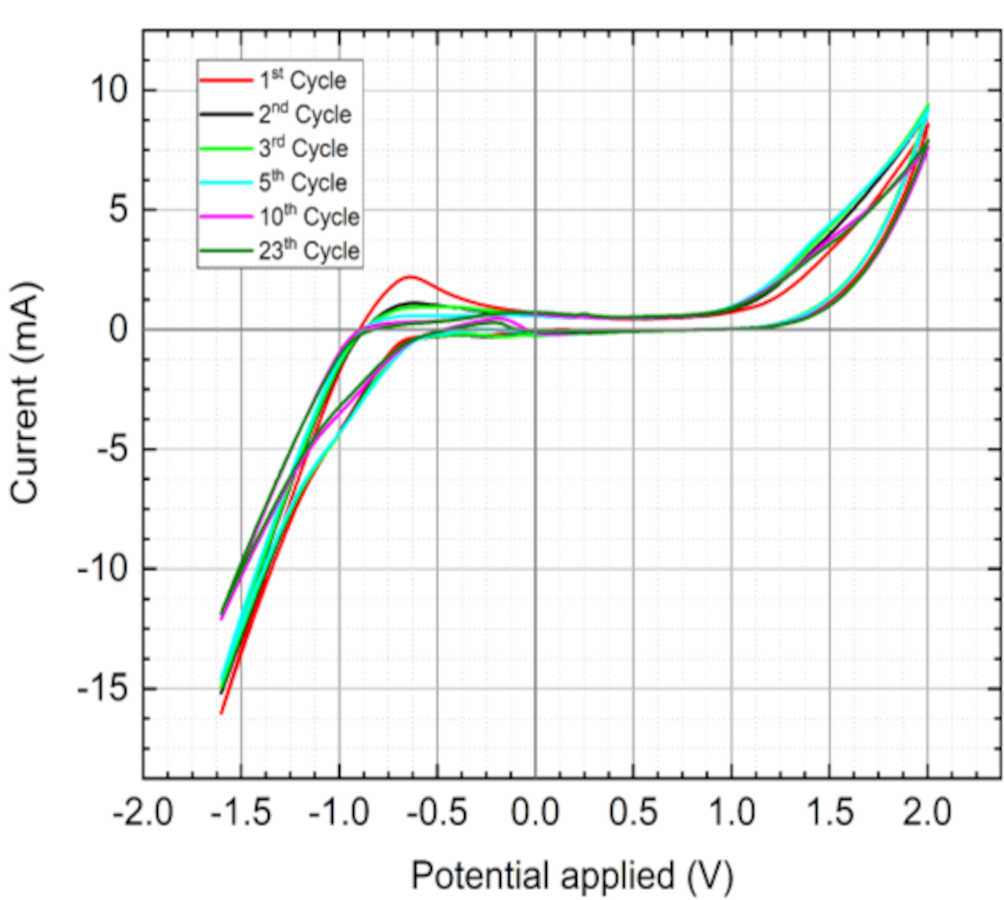

Supplement: Supplementary file 4 — Additional file 4: Fig. A4. Impact of electrode cycling on cyclic voltammetry (CV). CV was performed for up to 23 cycles. Note the change in current over the number of cycles. [file 13036_2023_393_MOESM4_ESM.tif]
